# Propane-1,3-diaminium bis­(pyridine-4-carboxyl­ate) monohydrate

**DOI:** 10.1107/S1600536811033502

**Published:** 2011-08-27

**Authors:** Iván Brito, Javier Vallejos, Alejandro Cárdenas, Matías López-Rodríguez

**Affiliations:** a Departamento de Química, Facultad de Ciencias Básicas, Universidad de Antofagasta, Casilla 170, Antofagasta, Chile; b Departamento de Física, Facultad de Ciencias Básicas, Universidad de Antofagasta, Casilla 170, Antofagasta, Chile; c Instituto de Bio-Orgánica ’Antonio González’, Universidad de La Laguna, Astrofísico Francisco Sánchez N°2, La Laguna, Tenerife, Spain

## Abstract

The asymmetric unit of the title compound, C_3_H_12_N_2_^2+^·2C_6_H_4_NO_2_^−^·H_2_O, consists of half of a doubly protonated propane-1,3-diammonium dication, a pyridine-4-carboxyl­ate anion and half of a solvent water mol­ecule; the dication and the solvent water are located on a twofold rotation axis which passes through the central C atom of the dication and the water O atom. The carboxyl­ate group of the anion appears to be delocalized on the basis of the C—O bond lengths. In the crystal, the components are linked by inter­molecular N—H⋯O, N—H⋯N and O—H⋯O hydrogen bonds.

## Related literature

For related compounds with a propane-1,3- diammonium dication which exhibit an all-*trans* zigzag conformation, see: Turner & Batten (2010 ▶); Aghabozorg *et al.*, (2011 ▶). For the preparation of the flexible ligand, see: Brito *et al.* (2010 ▶, 2011 ▶).
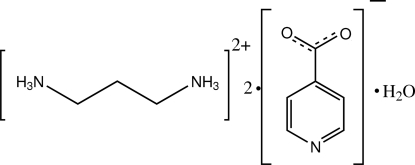


## Experimental

### 

#### Crystal data

C_3_H_12_N_2_^2+^·2C_6_H_4_NO_2_^−^·H_2_O
*M* *_r_* = 338.37
Monoclinic, 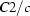

*a* = 15.360 (3) Å
*b* = 12.508 (3) Å
*c* = 10.593 (2) Å
β = 122.67 (3)°
*V* = 1713.2 (8) Å^3^
*Z* = 4
Mo *K*α radiation
μ = 0.10 mm^−1^
*T* = 295 K
0.27 × 0.25 × 0.21 mm

#### Data collection

Nonius KappaCCD area-detector diffractometer
11720 measured reflections
2119 independent reflections
1871 reflections with *I* > 2σ(*I*)
*R*_int_ = 0.050

#### Refinement

*R*[*F* ^2^ > 2σ(*F* ^2^)] = 0.046
*wR*(*F* ^2^) = 0.122
*S* = 1.06
2119 reflections
115 parameters
H atoms treated by a mixture of independent and constrained refinement
Δρ_max_ = 0.32 e Å^−3^
Δρ_min_ = −0.24 e Å^−3^

### 

Data collection: *COLLECT* (Nonius, 2000 ▶; cell refinement: *DENZO-SMN* (Otwinowski & Minor, 1997 ▶); data reduction: *DENZO-SMN*; program(s) used to solve structure: *SHELXS97* (Sheldrick, 2008 ▶ ); program(s) used to refine structure: *SHELXL97* (Sheldrick, 2008 ▶); molecular graphics: *OLEX2* (Dolomanov *et al.*, 2009 ▶); software used to prepare material for publication: *publCIF* (Westrip, 2010 ▶).

## Supplementary Material

Crystal structure: contains datablock(s) I, global. DOI: 10.1107/S1600536811033502/om2459sup1.cif

Structure factors: contains datablock(s) I. DOI: 10.1107/S1600536811033502/om2459Isup2.hkl

Supplementary material file. DOI: 10.1107/S1600536811033502/om2459Isup3.cml

Additional supplementary materials:  crystallographic information; 3D view; checkCIF report

## Figures and Tables

**Table 1:** Hydrogen-bond geometry (Å, °)

*D*—H⋯*A*	*D*—H	H⋯*A*	*D*⋯*A*	*D*—H⋯*A*
N2—H2*A*⋯O1^i^	0.89	1.91	2.7901 (16)	170
N2—H2*B*⋯O2^ii^	0.89	1.91	2.779 (2)	165
N2—H2*C*⋯N1^iii^	0.89	2.00	2.877 (2)	166
O3—H3⋯O1^iv^	0.85 (2)	2.00 (3)	2.8426 (17)	169 (2)

Symmetry codes: (i) 
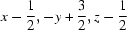
; (ii) 
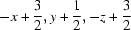
; (iii) 
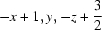
; (iv) 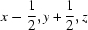.

**Fig. 1.**
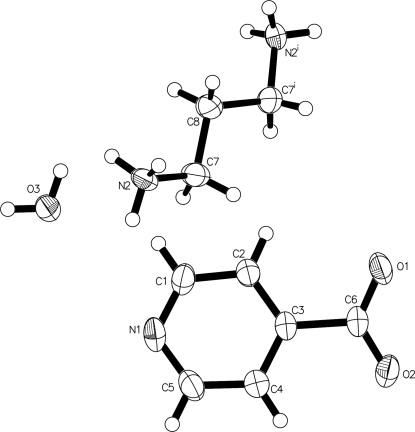
A view of the molecular structure of title compound, with the atom-numbering scheme. Displacement ellipsoids are drawn at the 50% probability level and H atoms are shown as small spheres of arbitrary radii. [Symmetry code i: 1 - x, y, 1/2 - z].

**Fig. 2.**
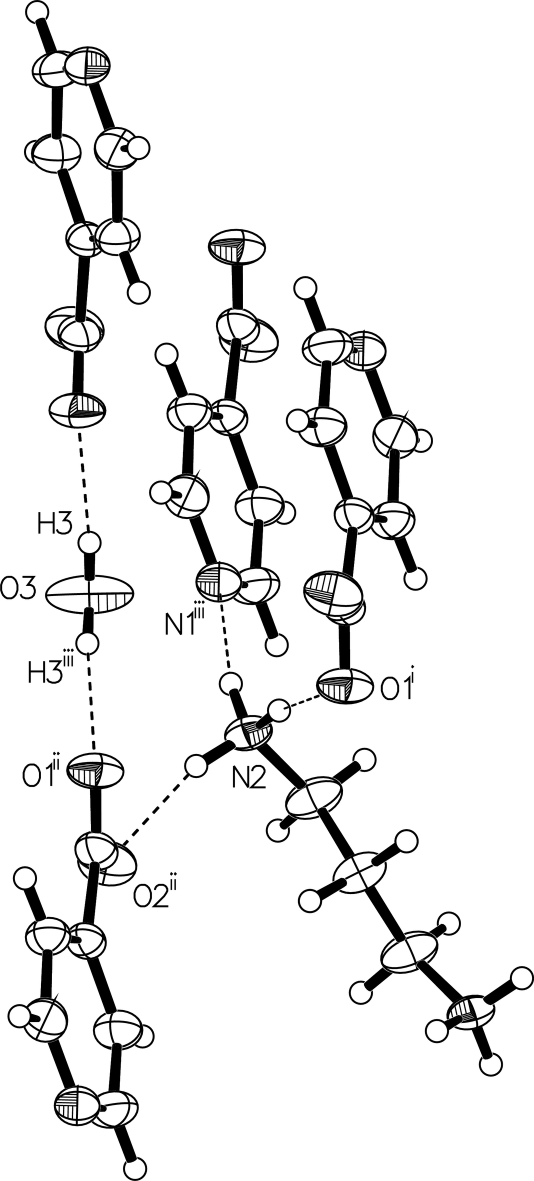
A view of title compound showing the hydrogen bonds and are indicated by dashed lines. [Symmetry codes: (i) x - 1/2, -y + 3/2, z - 1/2; (ii) -x + 3/2, y + 1/2, -z + 3/2; (iii) -x + 1, y, -z + 3/2; (iv) x - 1/2, y + 1/2, z]

## supplementary crystallographic information

### Comment

This paper forms part of our continuing study of the synthesis and structural characterization of a flexible ligand for preparation of coordination polymers (Brito *et al.*, 2010; 2011 and references therein). The title compound was isolated during attempts to synthesize a flexible ligand by a condensation reaction between 4-pyridinecarboxylic acid and 1,3-diaminopropane. A notable feature of the structure is the extensive network of the hydrogen bonds between the ammonium H and pyridine-carboxylate N, O atoms. The hydrogen-bonding network involves all of the ammonium H and pyridine-carboxylate O,*N* atoms, forming a three-dimensional network Fig.2, Table 1. The water H are linked to two carboxylate O atoms at (-1/2 + *x*, 1/2 + *y*, *z*) and at (3/2 - *x*,1/2 + *y*, 3/2 - *z*) respectively, forming a two-dimensional network. Propane-1,3-diammonium dication has a fully extended all-*trans* zigzag conformation (N/C/C/C torsion angle 167.57 (13)°). The bond distances and angles for dication and anion are in normal range (Aghabozorg *et al.*, 2011; Turner & Batten 2010, as representative examples).

### Experimental

To a solution of 4-pyridinecarboxylic acid (12.3 g, 0.1 mol) in pyridine (40 ml) was added 1,3-diaminopropane (3.71 g, 0,05 mol) in pyridine (20 ml). The solution was stirred gently for 15 min, forming a white precipitate. The resultant solution was then heated with stirring on the steam bath for 4 h. On cooling of the mixture, a white solid crystalline was obtained. Yield 15.8 g (92.9%). Analysis calculated for C_15_H_22_N_4_O_5_ (338.37 Dalton): C: 53.22, H: 6.52, N: 16.53; found: C: 52.97, H: 6.50, N: 16.90. IR (KBr, cm^-1^): (NH_3_^+^) 1664 m, (C=C) 1600 m, (NH_3_^+^) 1500 m, (CH_2_) 1378 w, (NH_3_^+^) 1155 m

### Refinement

H3 atom was located directly from a Fourier map and refined freely. The positions of the remaining H atoms were located from difference maps and then treated as riding atoms, with C—H distances in the range 0.93–0.97 Å and N—H distances of 0.89 Å and with *U*_iso_(H) values of 1.2*U*_eq_(C, N).

### Crystal data

**Table:**

C_3_H_12_N_2_^2+^·2C_6_H_4_NO_2_^−^·H_2_O	*F*(000) = 720
*M**_r_* = 338.37	*D*_x_ = 1.312 Mg m^−^^3^
Monoclinic, *C*2/*c*	Mo *K*α radiation, λ = 0.71073 Å
Hall symbol: -C 2yc	Cell parameters from 9511 reflections
*a* = 15.360 (3) Å	θ = 3.9–28.5°
*b* = 12.508 (3) Å	µ = 0.10 mm^−^^1^
*c* = 10.593 (2) Å	*T* = 295 K
β = 122.67 (3)°	Block, colourless
*V* = 1713.2 (8) Å^3^	0.27 × 0.25 × 0.21 mm
*Z* = 4	

### Data collection

**Table:**

Nonius KappaCCD area-detector diffractometer	1871 reflections with *I* > 2σ(*I*)
Radiation source: fine-focus sealed tube	*R*_int_ = 0.050
graphite	θ_max_ = 28.5°, θ_min_ = 4.0°
φ and ω scans with κ offsets	*h* = −20→20
11720 measured reflections	*k* = −16→16
2119 independent reflections	*l* = −14→11

### Refinement

**Table:**

Refinement on *F*^2^	Primary atom site location: structure-invariant direct methods
Least-squares matrix: full	Secondary atom site location: difference Fourier map
*R*[*F*^2^ > 2σ(*F*^2^)] = 0.046	Hydrogen site location: inferred from neighbouring sites
*wR*(*F*^2^) = 0.122	H atoms treated by a mixture of independent and constrained refinement
*S* = 1.06	*w* = 1/[σ^2^(*F*_o_^2^) + (0.0545*P*)^2^ + 1.0503*P*] where *P* = (*F*_o_^2^ + 2*F*_c_^2^)/3
2119 reflections	(Δ/σ)_max_ = 0.003
115 parameters	Δρ_max_ = 0.32 e Å^−^^3^
0 restraints	Δρ_min_ = −0.24 e Å^−^^3^

### Special details

**Table:**

Geometry. All s.u.'s (except the s.u. in the dihedral angle between two l.s. planes) are estimated using the full covariance matrix. The cell s.u.'s are taken into account individually in the estimation of s.u.'s in distances, angles and torsion angles; correlations between s.u.'s in cell parameters are only used when they are defined by crystal symmetry. An approximate (isotropic) treatment of cell s.u.'s is used for estimating s.u.'s involving l.s. planes.
Refinement. Refinement of *F*^2^ against ALL reflections. The weighted *R*-factor *wR* and goodness of fit *S* are based on *F*^2^, conventional *R*-factors *R* are based on *F*, with *F* set to zero for negative *F*^2^. The threshold expression of *F*^2^ > 2σ(*F*^2^) is used only for calculating *R*-factors(gt) *etc*. and is not relevant to the choice of reflections for refinement. *R*-factors based on *F*^2^ are statistically about twice as large as those based on *F*, and *R*- factors based on ALL data will be even larger.

### Fractional atomic coordinates and isotropic or equivalent isotropic displacement parameters (Å^2^)

**Table:**

	*x*	*y*	*z*	*U*_iso_*/*U*_eq_	Occ. (<1)
O1	0.87262 (8)	0.51431 (8)	0.82722 (12)	0.0447 (3)	
O2	0.81345 (8)	0.36420 (8)	0.86906 (13)	0.0480 (3)	
O3	0.5000	0.90541 (13)	0.7500	0.0712 (6)	
H3	0.4617 (17)	0.9452 (17)	0.766 (2)	0.072 (6)*	
N1	0.60893 (9)	0.65138 (10)	0.92600 (13)	0.0405 (3)	
N2	0.48117 (8)	0.79864 (8)	0.46730 (12)	0.0340 (3)	
H2A	0.4411	0.8559	0.4258	0.041*	
H2B	0.5458	0.8194	0.5334	0.041*	
H2C	0.4579	0.7600	0.5136	0.041*	
C1	0.64814 (10)	0.69083 (11)	0.84955 (15)	0.0389 (3)	
H1	0.6312	0.7605	0.8136	0.047*	
C2	0.71275 (10)	0.63306 (11)	0.82123 (14)	0.0355 (3)	
H2	0.7383	0.6636	0.7675	0.043*	
C3	0.73892 (9)	0.52889 (10)	0.87425 (13)	0.0301 (3)	
C4	0.69716 (10)	0.48635 (11)	0.95152 (16)	0.0386 (3)	
H4	0.7115	0.4163	0.9866	0.046*	
C5	0.63370 (11)	0.55061 (13)	0.97516 (17)	0.0437 (3)	
H5	0.6068	0.5220	1.0282	0.052*	
C6	0.81423 (9)	0.46330 (10)	0.85422 (14)	0.0339 (3)	
C7	0.47907 (13)	0.73319 (11)	0.34918 (17)	0.0431 (3)	
H7B	0.4119	0.6993	0.2884	0.052*	
H7A	0.5309	0.6773	0.3956	0.052*	
C8	0.5000	0.80121 (15)	0.2500	0.0401 (4)	
H8A	0.4408	0.8468	0.1876	0.048*	0.50
H8B	0.5592	0.8468	0.3124	0.048*	0.50

### Atomic displacement parameters (Å^2^)

**Table:**

	*U*^11^	*U*^22^	*U*^33^	*U*^12^	*U*^13^	*U*^23^
O1	0.0438 (5)	0.0454 (6)	0.0626 (7)	0.0006 (4)	0.0402 (5)	−0.0077 (5)
O2	0.0492 (6)	0.0347 (5)	0.0702 (7)	0.0085 (4)	0.0389 (6)	−0.0020 (5)
O3	0.0961 (14)	0.0306 (8)	0.146 (2)	0.000	0.1046 (16)	0.000
N1	0.0363 (6)	0.0498 (7)	0.0401 (6)	0.0113 (5)	0.0237 (5)	−0.0029 (5)
N2	0.0406 (6)	0.0330 (5)	0.0414 (6)	−0.0052 (4)	0.0306 (5)	0.0008 (4)
C1	0.0392 (7)	0.0383 (7)	0.0402 (7)	0.0127 (5)	0.0220 (6)	0.0027 (5)
C2	0.0361 (6)	0.0375 (7)	0.0393 (6)	0.0060 (5)	0.0246 (5)	0.0030 (5)
C3	0.0264 (5)	0.0332 (6)	0.0320 (6)	0.0027 (4)	0.0165 (5)	−0.0045 (4)
C4	0.0402 (7)	0.0371 (7)	0.0469 (7)	0.0049 (5)	0.0289 (6)	0.0036 (5)
C5	0.0438 (7)	0.0528 (8)	0.0483 (7)	0.0067 (6)	0.0340 (6)	0.0031 (6)
C6	0.0298 (6)	0.0366 (6)	0.0371 (6)	0.0048 (5)	0.0194 (5)	−0.0049 (5)
C7	0.0649 (9)	0.0301 (6)	0.0561 (8)	−0.0059 (6)	0.0469 (8)	−0.0024 (6)
C8	0.0597 (12)	0.0308 (9)	0.0484 (10)	0.000	0.0413 (10)	0.000

### Geometric parameters (Å, °)

**Table:**

O1—C6	1.2531 (16)	C2—H2	0.9300
O2—C6	1.2503 (17)	C3—C4	1.3896 (18)
O3—H3	0.85 (2)	C3—C6	1.5237 (15)
N1—C1	1.3373 (18)	C4—C5	1.3865 (18)
N1—C5	1.338 (2)	C4—H4	0.9300
N2—C7	1.4809 (17)	C5—H5	0.9300
N2—H2A	0.8900	C7—C8	1.5151 (16)
N2—H2B	0.8900	C7—H7B	0.9700
N2—H2C	0.8900	C7—H7A	0.9700
C1—C2	1.3851 (17)	C8—C7^i^	1.5151 (16)
C1—H1	0.9300	C8—H8A	0.9700
C2—C3	1.3897 (18)	C8—H8B	0.9700
			
C1—N1—C5	117.18 (11)	N1—C5—C4	123.75 (13)
C7—N2—H2A	109.5	N1—C5—H5	118.1
C7—N2—H2B	109.5	C4—C5—H5	118.1
H2A—N2—H2B	109.5	O2—C6—O1	126.17 (11)
C7—N2—H2C	109.5	O2—C6—C3	117.18 (11)
H2A—N2—H2C	109.5	O1—C6—C3	116.64 (12)
H2B—N2—H2C	109.5	N2—C7—C8	111.07 (11)
N1—C1—C2	123.24 (13)	N2—C7—H7B	109.4
N1—C1—H1	118.4	C8—C7—H7B	109.4
C2—C1—H1	118.4	N2—C7—H7A	109.4
C1—C2—C3	119.10 (12)	C8—C7—H7A	109.4
C1—C2—H2	120.5	H7B—C7—H7A	108.0
C3—C2—H2	120.5	C7^i^—C8—C7	111.68 (15)
C4—C3—C2	118.18 (11)	C7^i^—C8—H8A	109.3
C4—C3—C6	120.16 (12)	C7—C8—H8A	109.3
C2—C3—C6	121.62 (11)	C7^i^—C8—H8B	109.3
C5—C4—C3	118.52 (13)	C7—C8—H8B	109.3
C5—C4—H4	120.7	H8A—C8—H8B	107.9
C3—C4—H4	120.7		
			
C5—N1—C1—C2	−0.7 (2)	C3—C4—C5—N1	0.9 (2)
N1—C1—C2—C3	−0.1 (2)	C4—C3—C6—O2	20.19 (18)
C1—C2—C3—C4	1.19 (19)	C2—C3—C6—O2	−162.03 (12)
C1—C2—C3—C6	−176.62 (12)	C4—C3—C6—O1	−158.46 (13)
C2—C3—C4—C5	−1.6 (2)	C2—C3—C6—O1	19.31 (17)
C6—C3—C4—C5	176.26 (12)	N2—C7—C8—C7^i^	167.76 (14)
C1—N1—C5—C4	0.2 (2)		

Symmetry codes: (i) −*x*+1, *y*, −*z*+1/2.

### Hydrogen-bond geometry (Å, °)

**Table:**

*D*—H···*A*	*D*—H	H···*A*	*D*···*A*	*D*—H···*A*
N2—H2A···O1^ii^	0.89	1.91	2.7901 (16)	170
N2—H2B···O2^iii^	0.89	1.91	2.779 (2)	165
N2—H2C···N1^iv^	0.89	2.00	2.877 (2)	166
O3—H3···O1^v^	0.85 (2)	2.00 (3)	2.8426 (17)	169 (2)

Symmetry codes: (ii) *x*−1/2, −*y*+3/2, *z*−1/2; (iii) −*x*+3/2, *y*+1/2, −*z*+3/2; (iv) −*x*+1, *y*, −*z*+3/2; (v) *x*−1/2, *y*+1/2, *z*.
